# Self-Reported Prevalence and Awareness of Hyperhidrosis Among Medical Students at the Gulf Medical University, UAE

**DOI:** 10.7759/cureus.109385

**Published:** 2026-05-21

**Authors:** Abdulmalik M Garba, Sagr A H. Juma, Fauziyat K Adjoto, Salisu A Maikasuwa, Muhammad Q Bello, Jayadevan Sreedharan

**Affiliations:** 1 College of Medicine, Gulf Medical University, Ajman, ARE; 2 Epidemiology and Biostatistics, Gulf Medical University, Ajman, ARE

**Keywords:** awareness, hyperhidrosis, knowledge, medical students, prevalence

## Abstract

Introduction

Hyperhidrosis is a condition characterized by excessive sweating that can negatively affect quality of life and psychosocial well-being. This study aimed to determine the self-reported prevalence of hyperhidrosis symptoms and the level of awareness among medical students at Gulf Medical University, Ajman, UAE.

Methods

A cross-sectional study was conducted over six months among students aged ≥18 years across six academic programs. Ethical approval was obtained, and a pilot study was conducted before data collection. A structured self-administered questionnaire developed from a literature review and reviewed by specialists was used to assess sociodemographic characteristics, self-reported excessive sweating symptoms suggestive of hyperhidrosis, awareness, and associated factors. Data were analyzed using descriptive and inferential statistics.

Results

Out of 600 eligible participants, 480 completed the questionnaire (response rate: 80%). The majority were female (75.4%) and aged 18-21 years. The prevalence of self-reported excessive sweating symptoms suggestive of hyperhidrosis was 36.8% (171/465; 95% CI: 32.4%-41.2%). Among participants reporting excessive sweating symptoms who responded to the relevant items, the axilla was the most commonly affected site, followed by the face and hands. Afternoon was the most frequently reported time of excessive sweating. Awareness of hyperhidrosis was low, with only 31.7% of participants reporting prior knowledge, most commonly through the internet and academic sources. A significantly higher prevalence of self-reported excessive sweating symptoms was observed among males compared to females (p<0.01). Smoking was also significantly associated with self-reported excessive sweating symptoms (p<0.05). No significant associations were observed with age group, body mass index, or diabetes status. A proportion of participants reported a positive family history of similar symptoms.

Conclusion

Self-reported excessive sweating symptoms suggestive of hyperhidrosis were relatively common among medical students, but awareness remained low. While associations with gender and smoking were observed, the findings should be interpreted cautiously, given the use of self-reported data rather than clinically validated diagnostic criteria. Increased awareness and further studies using validated diagnostic instruments are recommended.

## Introduction

Hyperhidrosis is a condition characterized by excessive sweating beyond what is necessary for thermoregulation. It may be localized (primary focal hyperhidrosis) or generalized, and may be idiopathic or secondary to underlying medical conditions or medications. Diagnostic criteria for primary focal hyperhidrosis include focal, visible, excessive sweating of at least six months’ duration without apparent cause, and at least two of the following: bilateral and symmetric distribution, impairment of daily activities, frequency of at least once per week, age of onset <25 years, positive family history, and cessation during sleep [1].

Commonly affected areas include the axillae, palms, soles, face, and scalp. This condition significantly affects quality of life, particularly among young adults [2]. More recent studies have continued to highlight the under-recognition of hyperhidrosis and its psychosocial burden [3]. A 2013 European cohort study found that only 27% of individuals with clinically diagnosed hyperhidrosis had ever received medical evaluation or treatment for the condition, despite significant interference in social and occupational functioning [4].

The global prevalence of primary hyperhidrosis varies. Reported prevalence estimates differ considerably across studies because of variations in diagnostic criteria, assessment tools, and reliance on self-reported symptoms [5]. A study among medical students also demonstrated differences between subjective and objective assessment of hyperhidrosis, further emphasizing the influence of measurement methods on reported prevalence estimates [6]. A Japanese study reported a prevalence of 5.33% for palmar hyperhidrosis and 5.75% for axillary hyperhidrosis [7]. Similarly, a U.S. survey estimated the prevalence to be approximately 2.8%, with about 50% of individuals having axillary involvement [8].

Ethnic and regional differences have also been observed. A comparative analysis from Canada and China demonstrated similar prevalence rates (14.5% in Shanghai and 12.3% in Vancouver), suggesting minimal variation by ethnicity when adjusted for climate and lifestyle [9]. Consistent with previous studies, newer data indicate that obesity and BMI are associated with hyperhidrosis, although more research is needed to evaluate the role of weight reduction in symptom improvement [10].

Gender-related patterns remain inconsistent; while some studies suggest a higher prevalence among women, others find no significant difference [11]. Interestingly, another study from Europe found a higher likelihood of delayed diagnosis among males, potentially due to stigma or reduced healthcare-seeking behavior [12]. Family history, however, has been consistently linked with primary hyperhidrosis, suggesting a genetic predisposition [13,14].

Despite the potential influence of hot climate conditions on sweating-related symptoms in the Gulf region, published data on hyperhidrosis among university students in the UAE remain limited [15]. Medical students represent an important population because improved awareness and recognition of hyperhidrosis among future healthcare professionals may contribute to earlier diagnosis and better management of affected individuals.

The objectives of this study were: 1. To determine the self-reported prevalence of hyperhidrosis symptoms among medical students at Gulf Medical University, UAE. 2. To assess the level of awareness of hyperhidrosis among medical students. 3. To evaluate factors associated with self-reported hyperhidrosis symptoms.

## Materials and methods

A cross-sectional study was conducted among medical students at Gulf Medical University, Ajman, United Arab Emirates, over a six-month period. Eligible participants included students aged 18 years and above who were willing to participate and provided informed consent. Students who declined participation or did not provide consent were excluded from the study.

A population-based recruitment approach was used in which questionnaires were distributed to students across the six academic programs within the university, namely MBBS, Pharm D, BPT, DMD, BHS, and BBMS. The total eligible student population at the time of the study was approximately 600. Since the study aimed to recruit the entire eligible student population, no formal sample size calculation was performed. A total of 480 students completed the questionnaire, giving a response rate of 80%.

Data collection was conducted during school hours within classrooms and other areas of the university premises. Students were approached during breaks or free periods and were given sufficient time to complete the questionnaire voluntarily. Participation was entirely voluntary, and only students who agreed to participate were included in the study.

Data were collected using a structured self-administered paper questionnaire developed following a review of relevant literature and existing questionnaires on hyperhidrosis. The questionnaire consisted of five domains. The first domain assessed sociodemographic characteristics, including age, gender, nationality, body mass index (BMI), program, and year of study. The second domain evaluated factors associated with hyperhidrosis. The third domain assessed the self-reported prevalence of excessive sweating symptoms suggestive of hyperhidrosis. The fourth domain explored the psychosocial impact of excessive sweating and its interference with daily activities. The fifth domain assessed perceptions regarding treatment options for hyperhidrosis.

Participants were considered to have hyperhidrosis symptoms if they reported excessive sweating that interfered with daily activities. This self-reported operational definition was used because a validated diagnostic instrument, such as the Hyperhidrosis Disease Severity Scale (HDSS), was not utilized in this study.

The questionnaire underwent review by two specialists at Thumbay Hospital, Ajman, to assess clarity, relevance, and content appropriateness. Corrections and additional questions suggested during the review process were incorporated into the final version of the questionnaire. A pilot study was conducted among five students who were excluded from the final data collection. The pilot study was performed to assess the feasibility, clarity, ambiguity, and approximate completion time of the questionnaire.

Age was categorized into two groups (18-20 years and ≥21 years) for analysis. Participants who did not respond to specific questions were excluded only from analyses involving those variables; therefore, the total number of respondents varied across variables because of incomplete responses for some questionnaire items. Questions regarding anatomical site and timing of sweating permitted multiple responses, and analyses for these items were restricted to participants who provided valid responses.

Ethical approval for the study was obtained from the Gulf Medical University Ethics Committee on 24^th^ May 2016. Permission for data collection was also obtained from the university administration. Participation was voluntary, and anonymity and confidentiality were maintained throughout the study.

Data were entered into Microsoft Excel (Microsoft Corporation, Redmond, USA) and analyzed using Statistical Package for the Social Sciences (SPSS) version 23 (IBM Corp., Armonk, USA). Descriptive statistics were used to summarize participant characteristics and study findings. Associations between categorical variables were assessed using the chi-square test, and statistical significance was set at p < 0.05.

## Results

A total of 480 participants were included in the study, although response rates varied across variables because of incomplete responses to some questionnaire items. Among participants with available age data (n = 476), the majority were aged 19 years (25.8%), followed by participants aged 18 years and 20 years (23.9% each). Female participants constituted 75.4% of respondents (n = 463). Among participants with available BMI data (n = 435), most had normal weight (63.0%), followed by overweight (22.8%), underweight (8.7%), and obese (5.5%).

Among participants with available smoking data (n = 474), 12.4% reported smoking. Diabetes status was available for 380 participants, among whom 1.6% reported having diabetes mellitus, 91.6% reported no diabetes, and 6.8% had never checked their diabetic status. Most participants were from Asia (67.9%), followed by Africa (24.5%) (Table 1). Among 465 participants who responded to the relevant question, 171 reported excessive sweating interfering with daily activities, giving a self-reported prevalence of hyperhidrosis symptoms of 36.8% (95% CI: 32.4%-41.2%).

**Table 1:** Socio-demographic and Clinical Characteristics of Participants Total respondents varied across variables because of incomplete responses to some questionnaire items.

Variable	Group	Frequency (n)	Percent (%)
Age, years (n = 476)	18	114	23.9
	19	123	25.8
	20	114	23.9
	21	63	13.2
	22	33	6.9
	≥23	29	6.1
Gender (n = 463)	Male	114	24.6
	Female	349	75.4
BMI group (n = 435)	Underweight	38	8.7
	Normal weight	274	63.0
	Overweight	99	22.8
	Obese	24	5.5
Smoking status (n = 474)	Yes	59	12.4
	No	415	87.6
Diabetes status (n = 380)	Yes	6	1.6
	No	348	91.6
	Never checked	26	6.8
Nationality (by continent) (n = 473)	Asia	321	67.9
	Africa	116	24.5
	Europe	12	2.5
	South America	12	2.5
	North America	9	1.9
	Australia	3	0.6

The level of awareness regarding hyperhidrosis was assessed among participants, with 152 of 479 respondents (31.7%) reporting prior awareness of the condition. Among those aware of hyperhidrosis, the internet was the most frequently reported source of information (57.2%), followed by school or lectures (38.8%) and friends (32.9%). Other reported sources included family members, journals, television, and newspapers (Table 2).

**Table 2:** Awareness of Hyperhidrosis and Sources of Information Among Participants *Percentages for sources of information are calculated among participants who were aware of hyperhidrosis. Multiple responses were allowed.

Variable	Frequency (n)	Percent (%)
Awareness of hyperhidrosis (n = 479)		
Yes	152	31.7
No	327	68.3
Sources of information (n = 152)*		
Internet	87	57.2
School/lectures	59	38.8
Friends	50	32.9
Family	26	17.1
Journals	14	9.2
Television	12	7.9
Newspaper	11	7.2
Other sources	8	5.3

Among participants reporting symptoms suggestive of hyperhidrosis who provided responses regarding sweating sites, the axilla was the most commonly reported site of sweating (54.1%), followed by the face (48.6%) and hands (37.8%). Whole-body sweating and head/scalp involvement were also reported. Among participants without hyperhidrosis symptoms who responded to the same question, the axilla remained the most commonly reported site (62.0%) (Table 3).

**Table 3:** Distribution of Sweating Sites by Hyperhidrosis Status Percentages were calculated based on participants who provided valid responses to the sweating-site question. Multiple responses were allowed; therefore, percentages may exceed 100%.

Site of sweating	Hyperhidrosis Symptoms, Yes, n (%)	Hyperhidrosis Symptoms, No, n (%)
Armpit	20 (54.1%)	256 (62.0%)
Face	18 (48.6%)	139 (33.6%)
Hands	14 (37.8%)	87 (21.0%)
Feet	6 (16.2%)	43 (10.4%)
Head/scalp	8 (21.6%)	75 (18.2%)
Whole body	9 (24.3%)	48 (11.6%)
Other	0 (0.0%)	6 (1.6%)

Among participants reporting symptoms suggestive of hyperhidrosis who provided responses regarding the timing of sweating, the afternoon was the most frequently reported period of excessive sweating (54.1%), followed by morning and throughout-the-day sweating (24.3% each). Evening sweating was reported by 21.6% of participants, while night-time sweating was uncommon (2.7%). Among participants without hyperhidrosis symptoms who responded to the same question, sweating was also most commonly reported during the afternoon (77.4%) (Table 4). Overall, axillary involvement and afternoon predominance were the most consistent patterns irrespective of hyperhidrosis status.

**Table 4:** Distribution of Sweating Time by Hyperhidrosis Status Percentages were calculated based on participants who provided valid responses to the sweating-time question. Multiple responses were allowed; therefore, percentages may exceed 100%.

Time of sweating	Hyperhidrosis Symptoms, Yes, n (%)	Hyperhidrosis Symptoms, No, n (%)
Morning	9 (24.3%)	54 (12.9%)
Afternoon	20 (54.1%)	322 (77.4%)
Evening	8 (21.6%)	34 (8.2%)
Night	1 (2.7%)	25 (6.0%)
Throughout day	9 (24.3%)	31 (7.4%)

A positive family history of similar symptoms was reported by 143 participants (30.4%), while 328 participants (69.6%) reported no family history.

The prevalence of self-reported hyperhidrosis symptoms varied across participant characteristics (Table 5). A significantly higher prevalence was observed among males (49.5%) compared with females (33.1%) (p ≤ 0.01). Although participants aged 18-20 years demonstrated a higher prevalence than those aged ≥21 years (38.6% vs 31.7%), this difference was not statistically significant. No statistically significant association was observed between nationality and hyperhidrosis symptoms. Similarly, body mass index was not significantly associated with hyperhidrosis, although slightly higher prevalence estimates were observed among overweight/obese participants compared with normal-weight and underweight participants. Smoking status demonstrated a statistically significant association with hyperhidrosis symptoms, with smokers reporting a higher prevalence than non-smokers (50.9% vs 35.2%; p ≤ 0.05). No statistically significant association was identified between diabetes status and hyperhidrosis symptoms, although higher proportions were observed among participants with diabetes and those who had never checked their diabetic status.

**Table 5:** Association Between Hyperhidrosis and Participant Characteristics BMI: Body Mass Index

Variable	Group	Hyperhidrosis Symptoms, Yes, n (%)	Hyperhidrosis Symptoms, No, n (%)	Total	p-value
Gender	Male	53 (49.5)	54 (50.5)	107	≤0.01
	Female	113 (33.1)	228 (66.9)	341	
Age group, years	18–20	132 (38.6)	210 (61.4)	342	NS
	≥21	39 (31.7)	84 (68.3)	123	
Nationality (by continent)	Asia	121 (39.0)	189 (61.0)	310	NS
	Africa	40 (35.1)	74 (64.9)	114	
	Europe	5 (45.5)	6 (54.5)	11	
	South America	3 (25.0)	9 (75.0)	12	
	North America	1 (12.5)	7 (87.5)	8	
	Australia	0 (0.0)	3 (100.0)	3	
BMI group	Underweight	11 (30.6)	25 (69.4)	36	NS
	Normal weight	97 (36.5)	169 (63.5)	266	
	Overweight/Obese	46 (38.0)	75 (62.0)	121	
Smoking	Yes	29 (50.9)	28 (49.1)	57	≤0.05
	No	142 (35.2)	261 (64.8)	403	
Diabetes	Yes	4 (66.7)	2 (33.3)	6	NS
	No	112 (33.3)	224 (66.7)	336	
	Never checked	13 (50.0)	13 (50.0)	26	

## Discussion

This study demonstrates a relatively high self-reported prevalence of hyperhidrosis symptoms (36.8%; 95% CI: 32.4%-41.2%) among medical students at Gulf Medical University, contrasted with a notably low level of awareness (31.7%). This discrepancy highlights a significant gap between disease burden and recognition, even within a medically oriented population. This finding is comparable with a study in Brazil, which reported a prevalence of 20.6% among medical students, along with poor knowledge regarding the clinical definition and impact of hyperhidrosis [16], suggesting that hyperhidrosis remains underrecognized globally. Differences in diagnostic definitions, study populations, and the use of self-reported rather than clinically validated assessment tools may explain the substantially higher prevalence observed in the present study compared with clinically diagnosed cohorts. To our knowledge, published data assessing self-reported hyperhidrosis symptoms among university students in the UAE remain limited, making this study a useful regional contribution to the existing literature.

In terms of clinical characteristics, axillary involvement was the most commonly reported site of sweating, followed by the facial and palmar regions. These findings are consistent with existing literature identifying these areas as the most frequently affected in primary hyperhidrosis [2,7,8,16]. However, because this study relied on self-reported symptoms rather than validated diagnostic criteria, these findings should be interpreted cautiously. The predominance of afternoon sweating observed in this study may reflect environmental and climatic influences, particularly in warmer regions such as the United Arab Emirates. This pattern suggests that external factors, including ambient temperature and daily activity levels, may play a role in symptom exacerbation, consistent with findings from climate-related studies showing variation based on time of day and emotional stimuli [17].

A significantly higher prevalence of self-reported hyperhidrosis symptoms was observed among males compared to females in this study. This contrasts with earlier reports suggesting an equal gender distribution [4,16], but is in keeping with more recent regional findings that indicate potential gender differences in symptom perception and reporting [12]. These variations may be influenced by cultural, environmental, or behavioral factors. A proportion of participants reported a positive family history of similar symptoms, suggesting a possible familial tendency. This observation is in line with previous studies that have identified familial clustering and potential genetic associations in individuals with hyperhidrosis [14]. The predominance of female participants in the study population may also have influenced gender-related comparisons and should be considered when interpreting these findings.

With regard to associated factors, smoking was found to be significantly associated with self-reported hyperhidrosis symptoms, with a higher prevalence observed among smokers. Although this finding is not widely established in the literature, it may be related to the effects of nicotine on the autonomic nervous system, particularly its influence on sympathetic activity and sweat gland stimulation [18,19]. However, given the cross-sectional nature of this study, this association should be interpreted with caution and warrants further investigation in larger, longitudinal studies. In contrast, no significant association was observed between self-reported hyperhidrosis symptoms and BMI or diabetes status, suggesting that these factors may play a more prominent role in secondary hyperhidrosis than in self-reported sweating symptoms among otherwise healthy young adults [10,14,20].

Despite the relatively high self-reported prevalence observed, awareness of hyperhidrosis remained low among participants. Previous studies have highlighted the substantial psychosocial burden associated with hyperhidrosis, including impaired social interaction, emotional distress, and reduced quality of life [8]. This discrepancy may reflect differences in perception of symptom severity, cultural attitudes toward sweating, or limited awareness of the condition. It is also possible that participants may underreport the impact of symptoms due to normalization or a lack of recognition of hyperhidrosis as a medical condition. Increasing awareness among medical students may therefore improve recognition, reduce stigma, and encourage timely management [11,12,21]. Awareness of treatment options was not specifically assessed in this study; however, previous studies have suggested that individuals with hyperhidrosis often rely on lifestyle modifications and may delay seeking appropriate medical care [22].

This study has several limitations. The use of a self-reported questionnaire, rather than a validated diagnostic instrument such as the Hyperhidrosis Disease Severity Scale (HDSS), may have resulted in misclassification or overestimation of hyperhidrosis prevalence. Differences in diagnostic definitions and reliance on self-reported symptoms may partly explain the higher prevalence observed compared with clinically validated studies. Additionally, the cross-sectional design limits the ability to establish causal relationships. Variations in response rates across different variables and the presence of missing data may also have influenced the findings. The predominance of female participants may have introduced selection bias in gender comparisons. Finally, the study was conducted in a single institution, which may limit the generalizability of the findings.

## Conclusions

In conclusion, self-reported hyperhidrosis symptoms appear to be relatively common among medical students at Gulf Medical University, with a prevalence of 36.8% (95% CI: 32.4%-41.2%), yet awareness of the condition remained low (31.7%). Characteristic patterns such as axillary predominance and increased sweating during the afternoon were observed among participants who reported symptoms. A higher prevalence of self-reported hyperhidrosis symptoms was noted among males and smokers, while no significant associations were found with age, body mass index, or diabetes status. Given the reliance on self-reported symptoms rather than validated diagnostic criteria, the findings should be interpreted cautiously. Nevertheless, this study highlights the need for improved awareness, early recognition, and targeted education regarding hyperhidrosis among medical students, who may play an important role in identifying and managing affected individuals in future clinical practice.
